# Sex Differences in HIV Prevalence Persist over Time: Evidence from 18 Countries in Sub-Saharan Africa

**DOI:** 10.1371/journal.pone.0148502

**Published:** 2016-02-03

**Authors:** Hanne K. Hegdahl, Knut M. Fylkesnes, Ingvild F. Sandøy

**Affiliations:** Centre for International Health, University of Bergen, Bergen, Norway; Liverpool School of Tropical Medicine, UNITED KINGDOM

## Abstract

**Objective:**

The aim of this study was to examine changes over time in the female: male HIV prevalence ratio in 18 countries in Sub-Saharan Africa, overall and when stratified by area of residence, educational attainment and marital status.

**Methodology:**

We used data from the Demographic and Health Surveys, which are nationally representative household surveys. By using data from 18 countries with at least two survey rounds with HIV testing, and dividing the countries into three regions (Western/Central, Eastern and Southern) we were able to examine cross-country and regional changes in the female: male HIV prevalence ratio over time. Logistic regression was used to estimate female: male HIV prevalence ratios in urban versus rural areas and for different categories of education and marital status. To assess changes over time, we compared the confidence intervals of the prevalence ratios.

**Results:**

The female: male HIV prevalence ratio was above one in all countries in at least one survey round for both ages 15–24 years and 25–49 years. In 13 out of 18 countries the prevalence ratio was higher for the younger age group compared to the age group 25–49 years (3 significant) and this difference in prevalence ratios between the age groups did not change over time. Overall, there was a higher frequency of increasing than decreasing prevalence ratios. The gender disparity was greater among those who were married/living together than among the never-married, and over time, the ratio was more stable among the married/living together. The study found no clear differential changes by education.

**Conclusion:**

Women continue to carry the greater burden of HIV in Sub-Saharan Africa and there is no clear pattern of change in the gap between men and women as the direction and magnitude of change in the prevalence ratios varied greatly.

## Introduction

More than 30 years since it was first recognised, HIV continues to be a worldwide problem, with sub-Saharan Africa (SSA) being the region most severely affected by the epidemic. At the end of 2013, 35 million people were living with HIV globally, 24.7 million of which live in SSA [1]. Despite recent encouraging trends with declining HIV incidence in most SSA countries, gaps between different groups still remain, especially between men and women. In other regions of the world, more men than women are infected with HIV because epidemics are concentrated among men who have sex with men, injecting drug users, and sex workers and their male clients [1, 2]. In SSA however, heterosexual transmission has been the primary route, and the epidemics have spread to the general population. It is estimated that women account for 58% of the people living with HIV (PLWH) in the region, a skewed distribution that has been existing for years, and women on average acquire HIV as much as 5–7 years earlier than their male peers [1].

There are several explanations for why women in SSA are more vulnerable to contracting HIV than men. Importantly, women have higher biological susceptibility to HIV and STIs than men because of a larger surface area of mucous membrane being exposed during sexual intercourse and because of hormonal suppression of the immune system in the female genital tract during the secretory stage of the menstrual cycle [3, 4]. In addition to the higher biological susceptibility of women to HIV, sociodemographic factors such as gender-based violence, age discrepancy in relationships and limited access to education are key elements placing women in SSA at increased risk of HIV [5, 6].

However, the gender gap is not the only example of differences in HIV prevalence between subgroups. Urban populations have for many years exhibited higher HIV prevalence than rural populations in most SSA countries [7–10], and high educated groups have higher HIV prevalence than less educated groups [9, 11]. Studies related to marital status and HIV prevalence have differing findings, but most studies find that the divorced or widowed have higher HIV prevalence than the married and never-married [12–15]. When looking at gender disparity in relation to marital status, a study using data from 20 countries in SSA collected in the period 2003–2008 found that the gender disparity in HIV prevalence was greater among those who were unmarried (never-married/divorced/widowed) compared to the married [16].

Several studies have found declining trends in HIV prevalence among young people in different subgroups in SSA in recent years [9, 12, 13, 17–20]. However, when declines in HIV prevalence occur, they do not necessarily take place at the same speed in all subgroups. Recent declines in HIV prevalence have been shown to be steeper for urban than rural areas [9, 17]. The HIV epidemic in the region has also gradually shifted from being more severe among the higher educated than among less educated groups [21, 22] to many countries experiencing declining prevalence among the higher educated and stable or increasing prevalence among less educated young people [9, 17, 18, 23, 24].

HIV prevalence does not only depend on incidence of HIV but also on mortality of HIV. As increasing numbers of PLWH are receiving antiretroviral therapy (ART) and can live substantially longer lives, the epidemic is likely to change. For many years, more women than men have been receiving ART and women adhere better to treatment than men [25, 26]. This may affect the gender disparity in HIV prevalence because of increased mortality due to HIV and AIDS among men compared to women [26, 27]. Similarly to what has been observed in educational subgroups, it is possible that the relative burden of HIV on men and women may shift over time, and declining incidence is a clear sign that the HIV transmission patterns are changing. Therefore, the aim of this descriptive study was to assess whether there have been changes over time in the female: male HIV prevalence ratio in 18 countries in SSA, overall and when stratified by area of residence, educational attainment and marital status. Knowledge on changes in gender disparities is important for policy makers to establish whether there is a need for more targeted preventive interventions.

## Material and Methods

### Survey data sources

The analyses were based on data from the Demographic and Health Surveys (DHS) and the AIDS Indicator Surveys (AIS) conducted in the period 2001 to 2014 in 18 countries in SSA (see Table 1 for a list of the countries included). We included countries with data from at least two survey rounds (three in the cases of Mali, Tanzania and Zambia) in order to assess changes over time. These surveys are nationally representative population-based surveys conducted on average every five years in several low and middle income countries. The data can be accessed free of charge from www.measuredhs.com.

**Table 1:** Countries included and details of survey, by region.

Region	Country	Year of survey	Sample size (HH)	Response rate (%)		HIV testing rate (%)		Frequency of male questionnaires (HH)
				Men	Women	Men	Women	
**Western/Central Africa**	**Burkina Faso**	2003	9 097	90.5	96.3	85.8	92.3	1/3
		2010	14 424	97.3	98.4	94.4	96.7	1/2
	**Cameroon**	2004	10 462	93.0	94.3	89.9	92.1	1/2
		2011	14 214	95.6	97.3	93.0	94.7	1/2
	**DR Congo**	2007	8 886	95.4	96.7	87.0	91.0	1/2
		2013–14	18 171	97.4	98.6	94.0	96.0	1/2
	**Côte d’Ivoire**	2005	4 368	87.5	89.8	76.3	79.1	all
		2011–12	9 686	90.5	92.7	78.4	85.3	1/2
	**Guinea**	2005	6 282	94.5	97.2	88.2	92.5	1/2
		2012	7 109	96.7	98.0	94.4	96.6	1/2
	**Liberia**	2007	6 824	92.8	95.2	80.9	87.7	all
		2013	9 333	95.4	97.6	88.5	92.2	1/2
	**Mali**	2001	12 331	83.8	94.9	75.6	85.2	1/3
		2006	12 998	90.6	96.6	85.0	93.2	1/3
		2012–13	10 105	93.2	95.9	80.4	91.7	1/2
	**Niger**	2006	7 660	92.4	95.6	85.2	92.0	1/2
		2012	10 750	88.4	95.4	80.2	90.5	1/2
	**Senegal**	2005	7 412	86.0	93.7	75.5	84.5	1/3
		2010–11	7 902	87.0	92.7	78.5	86.0	1/3
	**Sierra Leone**	2008	7 284	92.6	94.0	86.7	89.5	1/2
		2013	12 629	96.4	97.2	90.2	98.9	1/2
**Eastern Africa**	**Kenya**	2003	8 561	85.5	94.0	70.3	76.3	1/2
		2008–09	9 057	88.6	96.3	79.4	86.4	1/2
	**Ethiopia**	2005	13 721	89.0	95.6	75.6	83.4	1/2
		2011	16 702	88.7	95.0	81.9	89.4	all
	**Rwanda**	2005	10 272	97.2	98.1	95.6	97.3	1/2
		2010	12 540	98.7	99.1	98.2	99.0	1/2
	**Tanzania**	2003–04	6 499	91.3	95.9	77.0	83.5	all
		2007–08	8 497	87.9	96.0	79.8	89.5	all
		2011–12	10 040	89.0	96.0	79.3	90.2	all
**Southern Africa**	**Zambia**	2001–02	7 126	88.7	96.4	80.1	81.1	1/3
		2007	7 164	91.0	96.5	72.2	77.1	all
		2013–14	15 920	91.1	96.2	83.7	90.4	all
	**Zimbabwe**	2005–06	9 285	81.9	90.2	63.4	75.9	all
		2010–11	9 756	85.8	93.3	69.9	80.6	all
	**Lesotho**	2004	8 592	84.6	94.3	68.0	80.7	1/2
		2009	9 391	95.0	97.9	88.1	93.8	1/2
	**Malawi**	2004	13 664	85.9	95.7	63.3	70.4	1/3
		2010	24 825	92.2	96.9	84.1	90.9	1/3

HH: households

The surveys were based on two-stage stratified cluster sampling. In the first stage, clusters from all regions of the countries were selected with probability proportional to size, then a pre-set number of households were randomly selected from the clusters. All women of reproductive age (15–49 years) were asked for an interview, and the same were men (aged 15–49, 15–54 or 15–59 years) in either all, every second or every third household (Table 1). It was possible to link HIV test results to the individual interviews and thus study sociodemographic factors associated with HIV.

Men and women in the households selected for interviews of men were asked to consent to blood specimens being drawn for HIV testing. All the specimens were tested with an ELISA test. With the exception of the last survey round in Zambia, where consenting individuals were offered an additional rapid HIV test in the household, HIV testing was anonymous and hence, the participants could not be provided with the test results. Instead they were given educational materials and referred to free voluntary counselling and testing (VCT). In places where distance to the VCT clinics was more than 15 kilometres, mobile VCT teams followed the interviewers.

### Statistical analyses

The analyses were conducted using Stata 13. They were adjusted for the survey design using the clustered robust option in Stata and sampling weights were applied to obtain nationally representative estimates. Indeterminate HIV test results were not taken into account and we did not distinguish between HIV-1 and HIV-2 positive status. Individuals with a valid HIV test result and information on age, sex and place of residence (urban/rural) were included in the analysis. All analyses were stratified by sex and place of residence, and conducted for two age categories (15–24 years and 25–49 years). A separate age category for young people was chosen because HIV prevalence in this age group can be used as a proxy for incidence. Men above 49 years of age were excluded in order to match the age distribution of the female participants. We adjusted for age as a continuous variable in the analyses of young people aged 15–24 years and as a categorical variable for the age group 25–49 years.

Crude and age-adjusted log-binomial regression was used to estimate HIV prevalence ratios between men and women in urban versus rural areas, at two educational levels (low versus high) and for two different categories of marital status (never married versus married/living together). To assess if there had been any changes in the prevalence ratio between the two (three) rounds of surveys, we added an interaction term between sex and time to the regression analyses. For Mali and Zambia it was not possible to link the first survey round to the latter rounds because the first rounds did not include clusters, thus we used comparison of confidence intervals to assess changes over time for these two countries. In the text we focus on the adjusted results unless there were any substantial differences between the crude and the adjusted analysis.

The analyses including education and marital status were only conducted for the age group 15–24 years to capture differential changes in recent infections. In order to increase the power to detect changes over time, the latter analyses were done on data pooled for three regions (Western/Central, Eastern and Southern Africa). The first wave of surveys for Mali (2001) and Zambia (2001–02) were not included in the regional analyses since the HIV datasets from these surveys could not be linked to information such as education and marital status in the female and male questionnaires, instead we used data from the second and third wave of surveys for these countries. The DHS sampling weights are normalised by dividing each weight by the average of the initial weights so that the sum of normalised weights equal the sum of cases over the entire sample. These weights are country specific, therefore the country sampling weights were denormalised according to the following formula for the regional analysis:

 Denormalised weight=weight×(total number of women and men aged 15−49/54/59 years in the country at the time of survey)/(number of women and men aged 15-49/54/59 interviewed in the survey)

In countries or regions where the statistical software was unable to provide a female: male HIV prevalence ratio due to prevalence of 0% in either of the groups (male or female), we judged the relative difference of the prevalence ratios based on the estimates of the HIV prevalence.

### Ethical considerations

The DHS methodology has been approved by the ICF International Institutional Review Board (IRB) in the US. In addition, the DHS protocols have been approved by IRBs or ethical committees in the host countries (27–65). Before each interview and HIV test was conducted, an informed consent statement was read to the respondent. This statement emphasised that the respondents could refuse to answer questions, to decline testing for HIV and that they could terminate participation at any time. For minors, parental or guardian consent was obtained in addition to the respondents’ assent. Verbal informed consent was sought by the interviewer reading a prescribed statement to the respondent and recording in the questionnaire whether or not the respondent consented (or provided assent on behalf of minors). Then the interviewer signed his or her name attesting to the fact that he/she read the consent statement to the respondent. Interviews and biomarker testing were conducted as privately as possible, and by assigning each respondent’s data files with identifier numbers that were destroyed after data processing, the respondents’ identities and results from interviews and HIV testing were kept strictly confidential and anonymous.

## Results

### Characteristics of study participants and response rates

The response rate varied from 81.9 to 98.7% among men and from 89.8 to 99.1% among women, and of those eligible for HIV testing, the testing rate was > 70% except in Malawi, Zimbabwe and Lesotho (Table 1). The total sample sizes varied from 4368 to 24825 households. More detailed descriptions can be found elsewhere [28–66].

### Changes over time in HIV prevalence ratio by urban/rural residence

#### Each country separately, 25–49 years

Table 2 shows that the female: male HIV prevalence ratio was above 1 in both survey rounds and in both urban and rural areas in 11 countries. Overall, nine of 18 countries had higher female: male ratios in urban than rural sites in both survey rounds, and among the rest, seven had a ratio that was higher in urban sites in one of the survey rounds. However, most of these differences were insignificant.

**Table 2:** Age-adjusted female: male HIV prevalence ratio (25–49 years) by country and urban/rural residence.

Urban						Rural				
Country	Female		Male		PR (95% CI)	Female		Male		PR (95% CI)
	HIV+ (%)	n	HIV+ (%)	n		HIV+ (%)	n	HIV+ (%)	n	
Burkina Faso										
2003	5.82	466	5.08	370	1.18 (0.57–2.44)	1.38	2 019	2.11	1 230	0.65 (0.38–1.11)
2010	4.17	1 413	1.77	1 282	2.39 (1.32–4.34)	1.01	3 718	0.72	2 544	1.44 (0.87–2.40)
Cameroon										
2004	10.76	1 277	8.09	1 184	1.33 (1.05–1.68)	5.77	1 530	4.18	1 343	1.38 (1.04–1.84)
2011	9.50	1 917	5.18	1 659	1.83 (1.38–2.44)	5.98	2 197	4.27	1 860	1.40 (1.11–1.77)
DR Congo										
2007	3.68	1 223	1.56	983	2.42 (1.36–4.31)	1.41	1 458	0.37	1 331	3.76 (1.37–10.4)
2013–14	3.47	1 831	0.98	1 422	3.63 (1.93–6.82)	1.27	3 718	0.58	3 035	2.28 (1.22–4.29)
Côte d´Ivoire										
2005	11.54	1 027	5.45	805	2.13 (1.35–3.34)	8.52	1 504	4.17	1 458	2.05 (1.29–3.26)
2011–12	8.25	1 084	5.26	911	1.61 (1.15–2.23)	4.68	1 678	3.24	1 449	1.46 (1.01–2.12)
Liberia										
2007	3.11	1 628	3.08	1 195	1.03 (0.65–1.62)	1.37	2 374	0.76	2 098	1.77 (0.96–3.25)
2013	4.34	942	3.91	804	1.11 (0.64–1.93)	1.50	1 774	0.84	1 620	1.78 (0.80–3.97)
Guinea										
2005	5.81	607	1.46	401	4.10 (1.72–9.80)	1.02	1 882	1.08	1 034	0.93 (0.44–1.95)
2012	5.47	934	2.24	737	2.44 (1.33–4.48)	1.39	1 856	1.54	1 165	0.92 (0.55–1.52)
Mali										
2001	4.10	522	3.11	401	1.32 (0.85–2.07)	2.00	1 817	1.51	1 203	1.28 (0.95–1.73)
2006	2.93	891	1.88	697	1.58 (0.86–2.90)	1.54	1 924	0.88	1 339	1.68 (0.74–3.82)
2012–13	2.68	871	2.71	538	1.03 (0.53–1.99)	1.12	2 402	0.67	1 643	1.70 (0.76–3.80)
Niger										
2006	2.48	845	2.46	612	1.00 (0.47–2.14)	0.46	1 912	0.71	1 118	0.66 (0.22–2.03)
2012	1.56	873	0.92	596	1.72 (0.72–4.13)	0.28	2 465	0.46	1 444	0.68 (0.28–1.62)
Senegal										
2005	1.32	1 021	0.79	665	1.68 (0.77–3.67)	1.13	1 454	0.81	801	1.46 (0.58–3.65)
2010–11	1.50	1 169	0.65	804	2.26 (0.67–7.68)	0.91	1 989	1.02	1 152	0.95 (0.52–1.71)
Sierra Leone										
2008	3.10	852	3.09	660	0.98 (0.56–1.71)	1.31	1 478	0.80	1 188	1.63 (0.67–3.98)
2013	2.88	1 705	3.01	1 382	0.95 (0.47–1.94)	1.34	3 066	0.87	2 366	1.55 (0.92–2.61)
Ethiopia										
2005	12.32	812	4.82	557	2.57 (1.63–4.05)	0.72	2 642	1.05	2 104	0.68 (0.34–1.35)
2011	9.35	2 524	5.03	2 009	1.89 (1.39–2.56)	1.10	6 543	0.70	5 083	1.58 (0.94–2.67)
Rwanda										
2005	12.45	691	9.44	548	1.34 (1.00–1.81)	3.84	2 514	2.71	1 783	1.43 (1.03–1.99)
2010	12.70	654	8.14	627	1.44 (1.08–1.92)	4.04	3 355	2.86	2 456	1.39 (1.08–1.80)
Kenya										
2003	16.10	547	11.15	463	1.43 (1.03–2.00)	9.15	1 310	6.01	1 026	1.54 (1.17–2.02)
2008–09	13.94	637	5.08	597	2.72 (1.58–4.66)	9.17	1 565	7.56	1 062	1.22 (0.93–1.59)
Tanzania										
2003–04	17.55	836	12.75	613	1.34 (1.02–1.77)	7.48	2 691	6.91	2 185	1.08 (0.86–1.36)
2007–08	14.46	1 106	10.19	686	1.43 (1.07–1.91)	6.72	4 020	6.27	2 836	1.07 (0.86–1.34)
2011–12	12.14	1 408	7.35	916	1.69 (1.18–2.43)	7.07	4 737	5.29	3 293	1.35 (1.12–1.63)
Lesotho										
2004	39.79	439	31.93	226	1.23 (0.92–1.64)	33.16	1 210	32.89	741	1.03 (0.90–1.18)
2009	39.44	563	33.72	319	1.18 (1.01–1.38)	35.92	1 569	30.47	1 074	1.17 (1.03–1.33)
Malawi										
2004	22.69	176	26.84	205	0.88 (0.60–1.28)	15.57	1 452	12.37	1 187	1.26 (1.03–1.54)
2010	31.26	570	20.47	453	1.52 (1.17–1.97)	15.23	3 768	11.26	3 015	1.37 (1.19–1.57)
Zambia										
2001–02	36.85	357	29.91	308	1.24 (1.02–1.51)	15.53	819	12.71	702	1.23 (0.98–1.53)
2007	32.72	1 358	24.71	1 099	1.35 (1.16–1.57)	13.83	1 987	13.26	1 719	1.04 (0.89–1.22)
2013–14	28.98	4 100	21.05	3 146	1.40 (1.26–1.56)	13.12	4 958	11.21	4 058	1.19 (1.07–1.31)
Zimbabwe										
2005–06	31.57	1 243	26.49	791	1.18 (1.02–1.36)	28.71	2 839	23.84	1 798	1.22 (1.10–1.34)
2010–11	27.92	1 508	19.35	950	1.41 (1.21–1.63)	23.76	3 095	19.21	2 176	1.23 (1.12–1.36)

PR: Prevalence ratio

**Fig 1:**
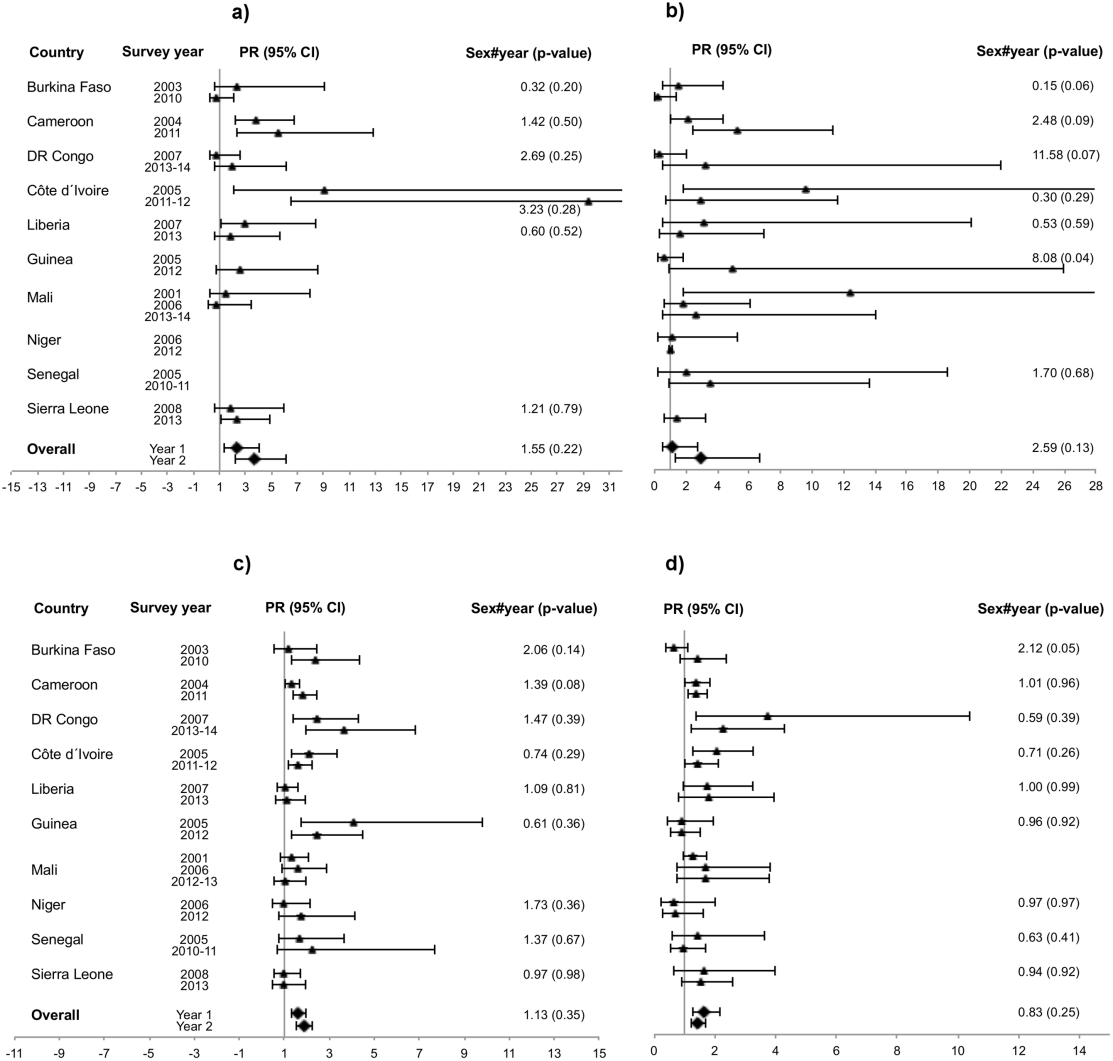
Western/Central Africa, age-adjusted female: male HIV prevalence ratios. (a) Urban, 15–24 years. (b) Rural, 15–24 years. (c) Urban, 25–49 years (d) Rural, 25–49 years.

**Fig 2:**
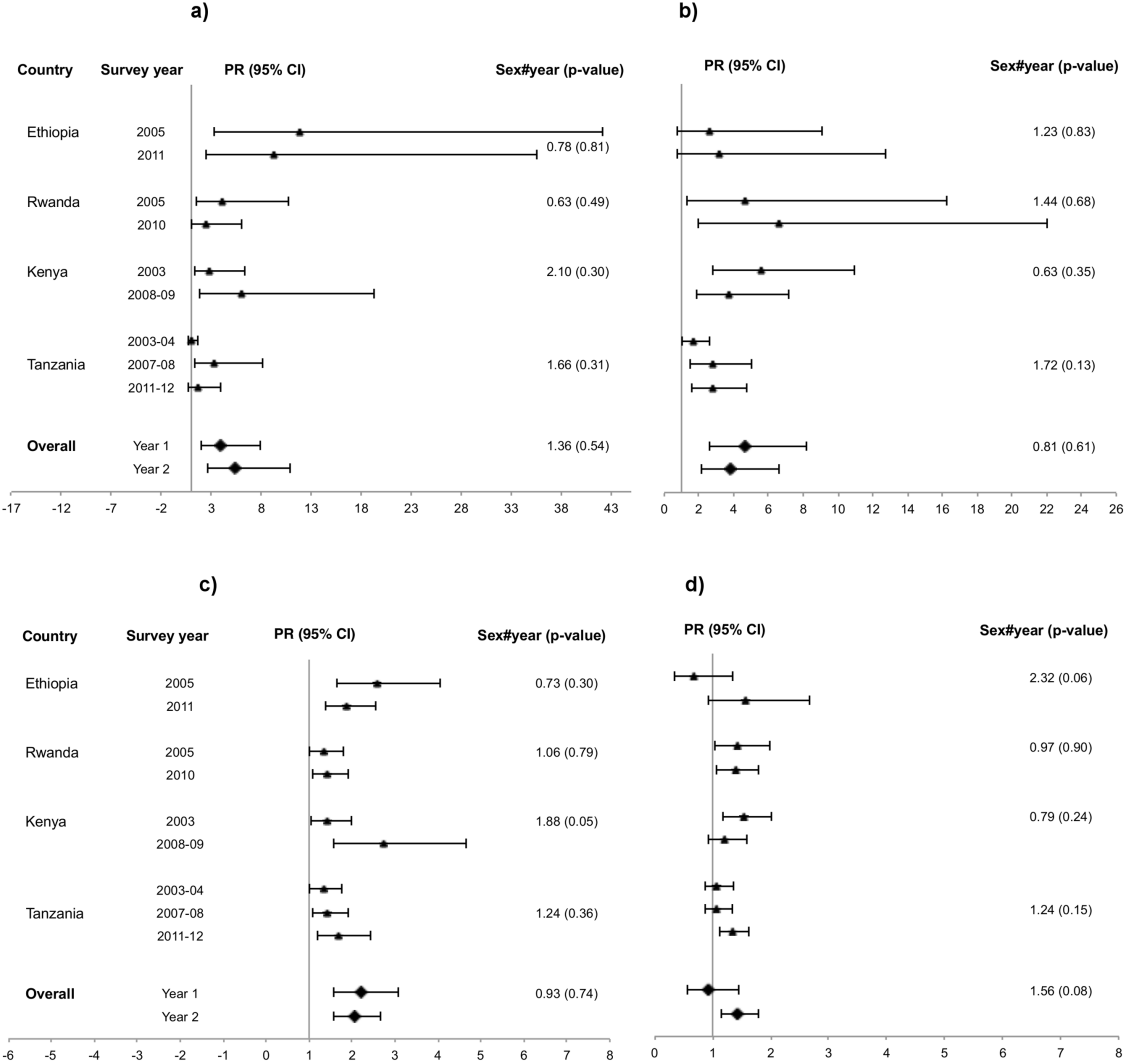
Eastern Africa, age-adjusted female: male HIV prevalence ratios. (a) Urban, 15–24 years. (b) Rural, 15–24 years. (c) Urban, 25–49 years (d) Rural, 25–49 years.

**Fig 3:**
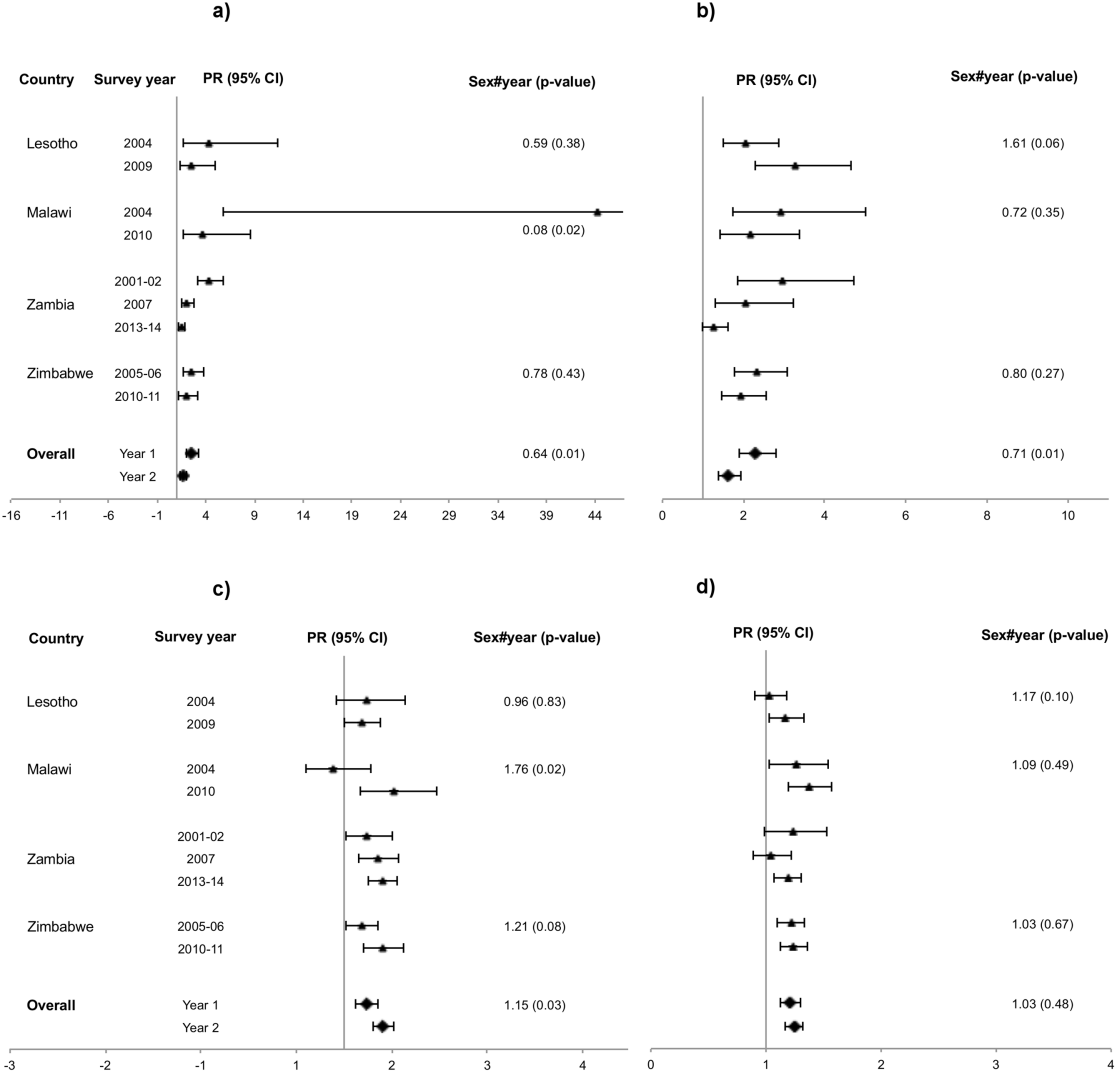
Southern Africa, age-adjusted female: male HIV prevalence ratios. (a) Urban, 15–24 years. (b) Rural, 15–24 years. (c) Urban, 25–49 years (d) Rural, 25–49 years.

Assessment of changes over time shows that in urban areas, the female: male prevalence ratio increased from the first to the last survey round in 12 countries and decreased in four countries (Figs 1c, 2c and 3c). However, the change was only significant in Malawi, where there was an increase from 0.88 to 1.52 (Fig 3c). In rural areas seven countries had increasing and nine had decreasing prevalence ratios but none of the changes were significant (Figs 1d, 2d and 3d). The remaining ratios were stable.

Zambia, Tanzania and Mali were the only countries with data from three points in time, making it possible to assess trends in the HIV prevalence ratios. Table 2 shows that in Mali, there was an overall decrease in the ratio in urban areas and an increase in rural areas. In Zambia, the ratio increased from 1.24 to 1.40 in urban areas, whereas in rural areas it decreased from the first to the second survey round, only to increase again in the third round (from 1.23 to 1.04 to 1.19). In Tanzania, the ratio increased in both urban and rural areas (from 1.34 to 1.69 and from 1.08 to 1.35, respectively, Fig 2c and 2d). None of the changes in either of the countries were significant.

#### Each country separately, 15–24 years

As with the older age group, the results for the age group 15–24 years (S1 Table) show that all countries had female: male HIV prevalence ratios above 1 in both urban and rural areas in at least one of the survey rounds. There were no significant differences in the prevalence ratios between urban and rural areas. In 13 out of 18 countries, the prevalence ratio was higher in this age group compared to the age group 25–49 years (3 of these differences were significant). The differences in prevalence ratio between the age groups did not change from the first to the last survey.

The female: male HIV prevalence ratio decreased significantly from the first to the second survey round in urban Malawi, whereas it increased significantly in rural Guinea (Figs 1b and 3a, respectively). Tanzania, Zambia and Mali showed differing trends. In Tanzania, there was an increase in the ratio from the first to the third round in both urban and rural areas (despite a decrease from the second to the third round in urban areas), however not significant (Fig 2a and 2b). In Zambia, there was a steady and significant decrease, both in urban and rural areas, over the three survey rounds. The decline was particularly steep in urban areas of Zambia, where the ratio decreased from 4.30 in the first round to 1.45 in the last round due to prevalence declines in females vs. an inverse time trend in males. Mali had no clear trends (S1 Table).

#### Regional, 25–49 years

When data for different countries was pooled at regional level (Table 3), the analysis showed that the female: male HIV prevalence ratio was higher in urban than in rural areas, however mostly insignificant. The only significant change over time was an increase in the ratio in urban Southern Africa (Fig 3c).

**Table 3:** Age-adjusted female: male HIV prevalence ratio by region and urban/rural residence.

Region	Urban					Rural				
	Female		Male		PR (95% CI)	Female		Male		PR (95% CI)
	HIV+ (%)	n	HIV+ (%)	n		HIV+ (%)	n	HIV+ (%)	n	
**Western and Central Africa**	25–49 years									
**1st round**	5.89	12 337	3–60	9 739	1.63 (1.34–1.99)	2.50	20 523	1.49	15 614	1.67 (1.28–2.17)
**2nd round**	4.96	12 739	2.67	10 135	1.87 (1.56–2.24)	1.79	24 863	1.28	18 278	1.44 (1.20–1.72)
**Eastern Africa**										
**1st round**	13.84	2 050	8.28	1 568	2.20 (1.56–3.09)	3.25	6 466	2.51	4 913	0.92 (0.58–1.45)
**2nd round**	10.74	3 815	5.29	3 233	2.05 (1.58–2.67)	3.12	11 463	2.18	8 601	1.43 (1.15–1.79)
**Southern Africa**										
**1st round**	31.78	3 216	25.87	2 321	1.23 (1.12–1.36)	21.08	7 488	17.45	5 445	1.21 (1.13–1.30)
**2nd round**	29.24	6 741	20.92	4 868	1.40 (1.30–1.52)	17.25	13 390	13.91	10 323	1.25 (1.17–1.32)
**Western and Central Africa**	15–24 years									
**1st round**	2.23	10 505	0.94	8 548	2.38 (1.39–4.07)	1.02	12 385	0.86	9 556	1.16 (0.49–2.75)
**2nd round**	1.48	10 606	0.39	8 376	3.67 (2.21–6.11)	1.02	14 104	0.33	10 362	2.93 (1.28–6.71)
**Eastern Africa**										
**1st round**	4.84	1 836	1.25	1 308	3.99 (2.01–7.89)	1.94	4 526	0.40	3 891	4.64 (2.62–8.20)
**2nd round**	1.82	3 248	0.35	2 112	5.43 (2.70–10.90)	1.32	7 754	0.34	6 496	3.78 (2.16–6.63)
**Southern Africa**										
**1st round**	11.61	2 884	4.62	2 098	2.53 (1.97–3.24)	9.17	5 519	3.62	4 477	2.31 (1.90–2.82)
**2nd round**	10.57	5 169	6.38	3 940	1.63 (1.33–2.01)	5.37	9 232	3.09	8 001	1.64 (1.38–1.95)

PR: Prevalence ratio

#### Regional, 15–24 years

The regional results for the age group 15–24 years showed that in Southern Africa there was a significant decrease over time in the HIV prevalence ratio in both urban and rural areas (Table 3 and Fig 3a and 3b). The HIV prevalence ratio was higher in the age group 15–24 years compared to the age group 25–49 years in all three regions, significantly so in Eastern and Southern Africa. In the age group 25–49 years, the ratio ranged from 0.92 to 2.20, whereas in the younger age group it ranged from 1.16 to 5.43.

### Changes in HIV prevalence ratio by marital status

#### Regional, 15–24 years

Analysis among 15–24 year olds showed that the ratio tended to be higher among those married/living together compared to the never married (S2 Table). In Southern Africa, the prevalence ratio significantly decreased among never-married from the first to the second survey round in both urban and rural areas.

### Changes in HIV prevalence ratio by education

#### Regional, 15–24 years

Analyses by educational level indicated few clear changes. The only region with significant changes in the HIV prevalence ratios by educational level was Southern Africa, where the ratios declined in the high education group in both urban and rural areas and in the low education group in urban areas (S3 Table).

## Discussion

This study shows that women continue to carry the greater burden of HIV in SSA with HIV prevalence ratios consistently being above 1, and there was no clear pattern of change in the gap between men and women as the direction and magnitude of change varied greatly. Furthermore, the gender disparity tended to be larger among young people than among older adults, indicating that young women are still the most vulnerable in the SSA HIV epidemic. For the age-group 25–49 years, there was a higher frequency of increasing prevalence ratios in urban areas compared to rural areas, however, mostly non-significant. The gender disparity was greater among those who were married or living together than among the never-married. Our study showed no clear differential changes in HIV prevalence ratios by education, indicating that women are more vulnerable than men independent of educational level. Of all our findings, the clearest changes were the ones seen among the youngest in Zambia where the prevalence ratios was decreasing, and in regions where the HIV prevalence was highest, such as in Southern Africa.

Overall, the female: male HIV prevalence ratio increased more often than it decreased. The increases were primarily due to a less marked prevalence decline among women compared to men, and reflected a larger increase in prevalence among women compared to men only in 8 cases in the age-group 25–49 years and in 5 cases for the age-group 15–24 years. Differential changes in sexual behaviour in both young and older age groups may explain the increases in prevalence ratios that we observed. Several studies from SSA investigating trends in sexual behaviour found that both women and men exhibited declining risky sexual behaviour [19, 67–69]. However, for most of the behavioural risk factors, such as sex before the age of 15 and 18 years, premarital sex and non-regular partners, the decline has been more marked among men than among women [19, 67–69]. It is thus plausible that the clearer declines in HIV prevalence among men may reflect bigger reductions in the rate of new infections among men than women. However, a study using data from three countries in SSA found that inequalities in HIV prevalence between men and women persisted even after the differential distribution of HIV risk factors and sociodemographic characteristics between men and women were controlled for [70], suggesting that biologic factors may be important in women’s greater vulnerability to HIV. Another possible explanation for the increasing prevalence ratios in some countries is the recent upscaling of voluntary medical male circumcision as a prevention strategy against HIV. Male circumcision may contribute to an increase in gender inequalities in HIV as it, on a short term basis, only protects men. Incidence of HIV in women can only decrease as an indirect result of medical male circumcision when the prevalence among men has fallen, which might take a long time to occur, and it can therefore not be regarded as a prevention strategy for women. Increasing ratios could also be due to lower HIV related mortality among infected women than men. Data from several countries indicate that more women than men are tested for HIV, that women have higher CD4+ counts when initiating ART compared to men, more women than men receive ART and women tend to adhere better to treatment [25, 71, 72]. These differences are likely due to the good availability of HIV testing and treatment services in antenatal settings [25, 71, 72]. Better survival with HIV infection can lead to higher HIV prevalence among women compared to men.

Power inequality in relationships, arising from unequal access to resources, decision-making on sexual and reproductive issues traditionally being male-controlled and substantial age differences between women and their male partners, is not uncommon in SSA and places women in a socially disadvantaged position with increased risk of HIV infection [25, 73]. The greater prevalence ratio that we found among young people compared to older adults and among young married or cohabiting people compared to young people who had never been married could be explained by age-disparate relationships as young women in SSA tend to marry men that are substantially older than themselves [74]. This increases young women’s risk of acquiring HIV by connecting to an older sexual network, and the largest age gaps have been found to occur in marriages and other long-term, stable relationships [74, 75].

In the analysis of change over time, we found that the prevalence ratio was more stable among the married than among the never-married. If we expect that most married couples engage in sexual intercourse on a regular basis and that never-married display greater variation in sexual activity, it is likely that the risk of acquiring HIV is more stable in married couples. It therefore seems logical to expect the ratio to change less in this group than in the never-married group. In support of our findings, a 2008 study from Rwanda and Zambia by Dunkle et al. [76] found that the risk of HIV transmission is higher within marriage or in cohabiting relationships than within non-cohabiting relationships.

We were only able to assess trends in the female: male HIV prevalence ratio in Mali, Tanzania and Zambia. Compared to the countries with data from two points in time, these three countries had clearer changes, however, we were only able to distinguish significant trends in Zambia. Among young people in Zambia there was a decrease in the prevalence ratio due to prevalence declines for women and increasing prevalence for men. In Tanzania there was a decrease in the HIV prevalence among both men and women, but due to a less marked decrease in the prevalence among women compared to men, the prevalence ratio increased in both age groups. It is difficult to find explanations for the trends in Zambia and Tanzania, and we could not find differential changes in sexual behaviour to help explain them. In Mali there were no clear trends.

A major strength of this study was the use of large datasets that have been collected in a standardised manner which make them fairly comparable across countries and time points. To our knowledge, no previous study has examined changes in gender differences over time in so many countries in SSA, and this gave us a good opportunity to look for overall patterns at regional and sub-regional level. The response rates were high with HIV testing rates above 80% in 8 countries and above 70% in 15 countries. Non-participation and non-testing was consistently higher among men than women, and data from the reports suggest this was due to men having more frequent and longer trips away from home than women [28–66]. Those who stay away from home because they are migrant workers have in other studies from SSA been found to be at higher risk of HIV infection [77, 78]. However, of the people who did not get tested, there were more people who refused to get tested than who were absent, and we cannot exclude the possibility that the ones who refused testing had a higher risk of being infected. Thus the differential HIV testing rates may have led to an overestimation of the female: male prevalence ratios, but due to the high testing rates it is not likely that this had substantial impact on our results. Reporting bias, which can often be a concern with DHS data because the interviews are very long (particularly for women) and there are many sensitive questions, is not likely to have had an impact on the data we analysed since we only used data on age, sex, education and marital status, which usually are not regarded as sensitive information.

A limitation of the analyses was that most of the countries we included only had data on HIV prevalence from two points in time, which made it impossible to assess time trends in these countries. In addition, pooling of data from different countries masked some of the differential changes at country level within a region. It is also worth noting that the time intervals were not the same for all countries within a region. Thus changes in the prevalence ratios at regional level should be interpreted with caution. However, the advantage with pooling was that some countries had a very small proportion of HIV positive individuals in some of the subgroups, giving very wide confidence intervals for the prevalence ratios, and pooling of data gave more power to assess changes over time. Another limitation to our study was that we only had prevalence data rather than incidence data. However, prevalence changes in young people can provide indications of incidence changes, thus we focused more on the age group 15–24 in our analyses.

## Conclusion

The continued vulnerability of women to HIV that we found in this study may indicate that biologic factors are important in explaining the higher HIV prevalence among women than among men and interventions need to have a stronger focus on tailored strategies for men and women. Women in SSA also face social challenges that should be reduced so that women may have the opportunity to protect themselves from HIV. Combining interventions that focus on changing the socioeconomic vulnerabilities of women with strategies increasing availability of ART and HIV testing services for men, may help eliminate the gender disparity in HIV prevalence. In conclusion it is worth pointing out the great heterogeneity of these data, which challenged the interpretation of the results. We have not, however, found any obvious or fitting explanations for this in SSA.

## Supporting Information

S1 Table Age-adjusted female: male HIV prevalence ratio (15–24 years) by country and urban/rural residence. (XLSX)

S2 Table Age-adjusted female: male HIV prevalence ratio by region, urban/rural residence and marital status (15–24 years). (XLSX)

S3 Table Age-adjusted female: male HIV prevalence ratio by region, urban/rural residence and educational level (15–24 years). (XLSX)

S4 Table Educational categories. (XLSX)
